# RURAL-URBAN DISPARITIES IN COMORBIDITY BETWEEN OBESITY AND DIABETES AMONG OLDER ADULTS

**DOI:** 10.1093/geroni/igac059.2406

**Published:** 2022-12-20

**Authors:** Youngjoon Bae

**Affiliations:** University of Massachusetts Amherst, Amherst, Massachusetts, United States

## Abstract

Obesity and diabetes are common among older Americans. While obesity is a risk factor for diabetes, research on rural-urban health disparities among older adults has reported paradoxical results. That is, living in rural areas is a risk marker of obesity, whereas rural residency is inversely associated with diabetes. This study utilized multiple national data resources to diagnose the inconsistency, including the 2006-2016 RAND HRS Longitudinal data, biomarker data, the 2010 US Census, and the USDA food access information. HRS survey participants 65 to 97 years of age comprised the study sample (N=6,476). General generalized estimating equation models with survey weights were used to estimate associations with measured obesity and diabetes. Age, race, Hispanic status, foreign-born status, education, marital status, working status, wealth, physical activity, smoking, drinking, ADL, IADL, loneliness, depression, cholesterol, HDL, CRP, and Cystatin C were included as control variables. Findings suggest that rurality is associated with a higher likelihood of obesity and a lower likelihood of diabetes, particularly for older women. However, by adjusting for confounders, these relationships disappeared. Instead, social isolation indicated significant associations with a lower likelihood of obesity and a higher likelihood of diabetes among older women. Contrary to prior research, the food desert and food insecurity measures did not correlate with both outcome variables. Further investigation on food consumption and dietary behavior among socially isolated older women is needed.

